# Application of Rice Straw Inhibits Clubroot Disease by Regulating the Microbial Community in Soil

**DOI:** 10.3390/microorganisms12040717

**Published:** 2024-04-01

**Authors:** Zhe Han, Yiping Zhang, Chengqian Di, Hongwen Bi, Kai Pan

**Affiliations:** 1 Institute of Agricultural Remote Sensing and Information, Heilongjiang Academy of Agricultural Sciences, Harbin 150086, China; hanzhe6615@aliyun.com (Z.H.); bhw01@126.com (H.B.); 2 Heilongjiang Academy of Agricultural Sciences Postdoctoral Program, Harbin 150086, China; 3 College of Horticulture and Landscape Architecture, Northeast Agricultural University, Harbin 150030, China; 18846054105@aliyun.com (Y.Z.); princedcq@sina.com (C.D.)

**Keywords:** rice straw, Chinese cabbage, clubroot, soil microbes, Illumina MiSeq

## Abstract

Straw return is an effective agricultural management practice for alleviating soil sickness, but only a few studies have focused on the incorporation of straw with deep plowing and rotary tillage practices in vegetable production. To determine the effects of rice straw return on Chinese cabbage clubroot, a field experiment for three consecutive years in the same area was performed. Soil microbial high-throughput sequencing, quantitative real-time polymerase chain reaction (PCR) and other methods were used to detect Chinese cabbage plant growth, clubroot occurrence, soil chemical properties and soil microbial diversity and abundance. The results showed that straw addition could significantly reduce the clubroot disease incidence. Through Illumina Miseq sequencing, the diversity of the fungi decreased obviously. The relative abundance of the phyla Proteobacteria and Firmicutes was strikingly reduced, while that of Chloroflexi was significantly increased. Redundancy analysis suggests that soil properties may also affect the soil microbial composition; changes in the microbial structure of bacteria and fungi were associated with the available phosphorus. In conclusion, the continuous addition of rice straw can promote the growth and control the occurrence of clubroot, which is closely related to the microbial composition, and the inhibition effect is proportional to the age of addition.

## 1. Introduction

Clubroot disease is a serious and widespread soil-borne disease that threatens cruciferous crop production and is caused by the obligate parasite *Plasmodiophora brassicae* [1]. Clubroot disease devastates both agricultural plants and field soil; about 3.2–4.0 hectares of cruciferous crops in China are affected by clubroot disease each year [2,3]. The development of resistant cultivars is considered the most important approach for controlling this plant disease, and genes have been identified in Chinese cabbage breeding. However, in disease control, several measures have been proposed to alleviate clubroot, but the effects of resistance breeding and chemical agents are not ideal [2]. The tenacious activity of *P. brassicae* in soil and its ability to overcome the genetic resistance of commercial hybrids make it a major threat to cruciferous crop production. Therefore, effective and safe prevention and control methods are particularly important.

The abundance of pathogens in soil is closely related to the occurrence of soil-borne diseases. Planting previous crops is one of the important measures to control soil-borne diseases, as it can create unfavorable environments to reduce crop damage by soil pathogens [4]. Studies have shown that non-host crops can reduce pathogen proliferation, but so far, the results have not been satisfactory [5]. The rotation of legumes and gramineous crops with cruciferous crops can alleviate the occurrence of cruciferous clubroot; these include soybeans, clover, wheat, rice, and maize [6,7]. However, crop rotation practices are limited by economic value, means of operation, and limited cropping options [8].

Crop straw is the oldest and most economical management method for alleviating monoculture problems and improving crop yield and quality, and it has been widely observed that crop straw is beneficial for reducing soil-borne pathogens. Straw returning to the field has great advantages in residue management and soil protection of crop planting systems and solves the problems of open burning and discarding. The straw represents the main nutritional elements of plants; proper returning of the straw to field can improve soil fertility [9]. However, the effect of grain crop straws on plant performance and the soil environment remains unclear so far.

Previous studies have shown that gramineous straw amendments could inhibit clubroot disease and promote the growth of Chinese cabbage, and rice straw worked better than maize and wheat straw [10]. In addition, exogenous straw application can not only effectively increase soil organic matter but also increase the soil carbon and nitrogen pool, promote changes in the soil microbial community, and improve the field ecosystem [11]. Repeated application of rice straw changed the soil chemical properties and altered the bacterial community composition to suppress the clubroot disease incidence in Chinese cabbage Repeated application of straw inhibited clubroot of Chinese cabbage by altering soil chemical properties and bacterial community composition [12]. Therefore, we aimed to investigate the effects of rice straw amendments on the performance and incidence of clubroot disease of Chinese cabbage through a 3-year consecutive field experiment. We hypothesized that the addition of rice straw could improve the plant growth of Chinese cabbage under continuous cropping and inhibit clubroot due to the improvement in the soil environment.

## 2. Materials and Methods

### 2.1. Study Area

A three-year field experiment with Chinese cabbage (*Brassica rapa pekinensis*) was carried out to evaluate the effect of straw amendment on the soil at a research site located in Baicheng Village, Acheng District, Harbin, China (126°59′ E, 45°29′ N) during 2017–2019. The location has a temperate continental monsoon climate, with an annual precipitation of 542 mm and a mean annual temperature of 4 °C; the average soil temperature at 5 cm underground during the test was 21 °C. The planting mode of spring garlic (*Allium sativum*) and autumn Chinese cabbage (*Brassica rapa pekinensis*) has been practiced for more than 30 years. In the last 10 years, the incidence of clubroot has been severe, and cultivated fallow and disease-resistant varieties had limited effectiveness.

The soil was black soil (Mollisol), and soil samples were collected to determine the chemical properties before planting Chinese cabbage in July 2017. The soil had a pH of 6.97, an electrical conductivity (EC) value of 0.140 mS/cm, a soil organic matter (SOM) content of 4.01%, an alkali hydrolyzable nitrogen (AN) content of 173.70 mg·kg^−1^, an available phosphorus (AP) content of 764.07 mg·kg^−1^, and an available potassium (AK) content of 516.00 mg·kg^−1^. The basic soil chemical properties before sowing in July 2019 are listed in Supplementary Table S1.

The variety of Chinese cabbage was Gailiangdongbai 1 (susceptible to *P. brassicae*), provided by the Cabbage Research Group of Northeast Agricultural University (Harbin, China). Rice (*Oryza sativa*) straw was collected from a traditional rice production field on the outskirts of Harbin, then naturally air-dried in the shade and cut into 3–5 cm pieces for later use. The organic matter content of the straw was 62.58%, the total nitrogen content was 0.76%, the total phosphorus content was 0.52%, the total potassium content was 3.20%, and the carbon–nitrogen ratio (C/N) was 82.74:1 [12].

### 2.2. Experimental Design

The study was conducted after the garlic harvest in July 2017. A randomized block design was adopted, with 12 plots selected for 4 treatments (MTR, MSR, MFR and MCK), with 3 replicates per treatment. For the three-year application (MTR; 3 plots), rice straw was added into the field after the garlic harvest in 2017, 2018 and 2019, separately. For the two-year application (MSR; 3 plots), rice straw was added into the field after the garlic harvest in both 2018 and 2019. For the one-year application (MFR; 3 plots), rice straw was added after the garlic harvest only in 2019. For the control (MCK; 3 plots), no rice straw was added in 2017, 2018 or 2019. The plot size for Chinese cabbage planting was 3 × 4 m per replicate, and the planting spacing was 60 × 60 cm. In the straw-addition treatments, 3.5 kg of rice straw was applied at a depth of 15 cm before sowing. Rotten cow dung (30 m^3^ ha^−1^) was applied every spring. Before the plantation of both garlic and Chinese cabbage, a compound fertilizer (N, 18%; P2O5, 46%; 450 kg ha^−1^) was added twice a year. Urea (375 kg ha^−1^) was applied in late August each year. Irrigation measures were taken in the cultivation process according to the rainfall situation.

### 2.3. Determination of Growth and Disease

Plant samples were collected in 2017, 2018 and 2019, separately. Sixty days after sowing, the fresh shoot weight was measured using a conventional weighing method. The incidence rate of clubroot was measured 30 days after sowing based on Walkley et al. [13].

### 2.4. Soil Collection and Chemical Property Analysis

Soil samples were collected in 2017, 2018 and 2019, separately. Forty days after sowing, soil was collected from a depth of 5–15 cm around the plants and sieved (2 mm) to remove visible impurities, and three soil samples were mixed to make a duplicate soil sample. The soil samples were transferred to the laboratory, and some of the partially fresh soil samples were stored in a refrigerator at −70 °C for DNA extraction and high-throughput sequencing 40 days after sowing.

The soil chemical properties were analyzed in the laboratory; the pH and EC were determined according to Walkley et al. [13], and AN, AP, SOM and AK were determined based on Bao et al. [14].

### 2.5. DNA Extraction and Quantitative Polymerase Chain Reaction (PCR) Analysis

Six duplicates of DNA were extracted from each treatment, for a total of 24 samples. Soil DNA was extracted using the PowerSoil DNA Isolation Kit (Mo Bio Laboratories Inc., Carlsbad, CA, USA) according to the manufacturer’s protocols. The abundance of *P. brassicae* in the soil was determined using an iQ5 Real-Time PCR Detection System (Bio-Rad Lab, Hercules, CA, USA), based on Wallenhammar et al. [15]. The forward primer was PbF (AAACAACGAGTCAGCTTGAATGC), and the reverse primer was PbR (TTCGCGCACAAGCACTTG). The number of resting spores was determined based on the concentration of *P. brassicae* in the soil DNA (y = 697.82x^0.9925^, R^2^ = 0.9983), which was calculated from the concentration of plasmid DNA [15].

### 2.6. Illumina MiSeq Sequencing and Data Processing

Soil bacterial and fungal community compositions were analyzed using Illumina MiSeq sequencing [16]. The 16S rRNA genes in the V3–V4 region of the bacteria were analyzed using primers 341F and 806R [17], and the ITS2 region of the fungi was analyzed using primers ITS3 and KYO2/ITS4 [18].

The soil DNA was amplified using triplicate polymerase chain reaction (PCR), and purified amplicons were received after detection and quantification. The Illumina platform was used for the paired-end sequences (PE250) according to standard protocols. The raw reads were further filtered and merged against the reference database, and the effective tags were clustered into operational taxonomic units (OTUs) with 97% similarity.

### 2.7. Statistical Analysis

The experimental data were analyzed with one-way analysis of variance (ANOVA) using Tukey’s honestly significant difference (HSD) test in SPSS software (Version 19.0, Armonk, NY, USA). The data means were considered significantly different at *p* < 0.05. The bar diagram was prepared using OriginPro software (Version 8.5, OriginLab Corporation, Northampton, MA, USA).

The sequenced data were analyzed using Quantitative Insights Into Microbial Ecology (QIIME), Version 1.9.0. For beta diversity, the bacterial and fungal community structures were analyzed by principal coordinates analysis (PCoA) with Bray–Curtis distance dissimilarity, and ANOSIM and Adonis were used to clarify the differences in the community compositions in the different treatments.

## 3. Results

### 3.1. The Plant Growth Response to the Straw Addition

Rice straw addition significantly increased the fresh weight of the aboveground parts compared with the control (*p* < 0.05). Rice straw addition treatments also significantly decreased the clubroot incidence rate compared with the control (*p* < 0.05). In addition, the incidence rate in the three-year application treatment was significantly higher than that in the one-year and two-year application treatments (Table 1).

### 3.2. The Soil Chemical Property Response to the Straw Addition

The rice straw addition significantly increased the available potassium content compared with the control (*p* < 0.05). The three-year addition significantly raised the content of alkaline hydrolytic nitrogen, available phosphorus, available potassium and total nitrogen compared with the control (*p* < 0.05). The two-year addition significantly increased the content of available phosphorus, available potassium and total nitrogen compared with the control (*p* < 0.05) (Table 2).

### 3.3. The Abundance of Plasmodiophora brassicae Response to the Straw Addition

**Figure 1:**
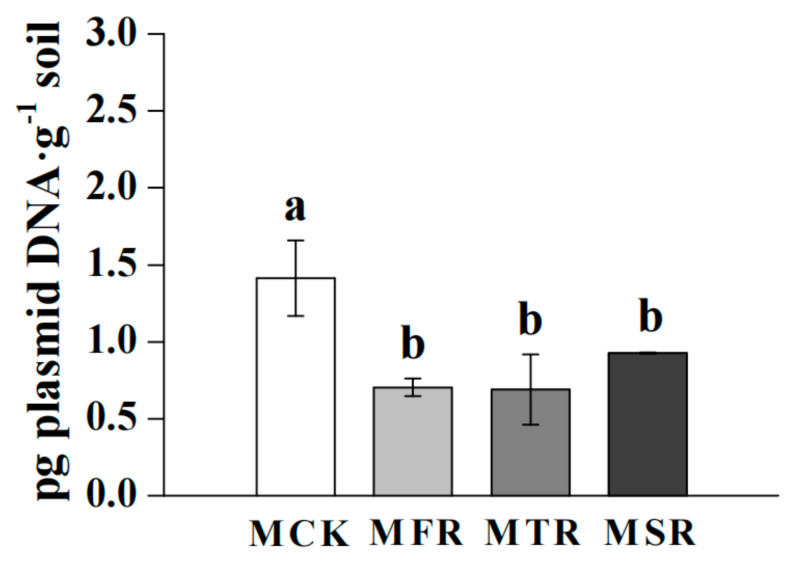
Abundance of *Plasmodiophora brassicae* in response to straw application. MCK represents no straw addition; MFR, MSR, and MTR represent one-year, two-year and three-year addition of rice straw, respectively. Error bars indicate the standard error, and different letters indicate significant differences at the 0.05 level (Tukey’s HSD test).

Forty days after sowing, the rice straw addition significantly reduced the abundance of resting spores compared with the control (*p* < 0.05) (Figure 1).

### 3.4. The Abundance of Bacterial and Fungal Responses to the Straw Addition

In the quantitative PCR results, the abundance of bacteria and fungi showed no significant difference between the rice straw addition and control (*p* < 0.05) (Supplementary Figure S1).

### 3.5. Alpha and Beta Diversities of Bacterial and Fungal Communities in the Soils

Twenty-four samples of bacterial 16SRNA V3-V4 regions and twenty-four fungal ITS regions were subjected to Miseq sequencing in the research. Across all soil samples, the Illumina Miseq sequencing generated 2,848,059 and 2,994,566 optimized sequences of bacteria and fungi, respectively. Good’s coverage reflects the captured diversity, with averages of 98.72% (ranging from 98.54% to 98.91%) for bacterial communities and 99.90% (ranging from 99.89% to 99.92%) for fungal communities. The rarefaction curves of bacteria and fungi are shown in Supplementary Figure S2; the number of sequences was adequate to represent the diversity of soil microbial communities.

**Figure 2:**
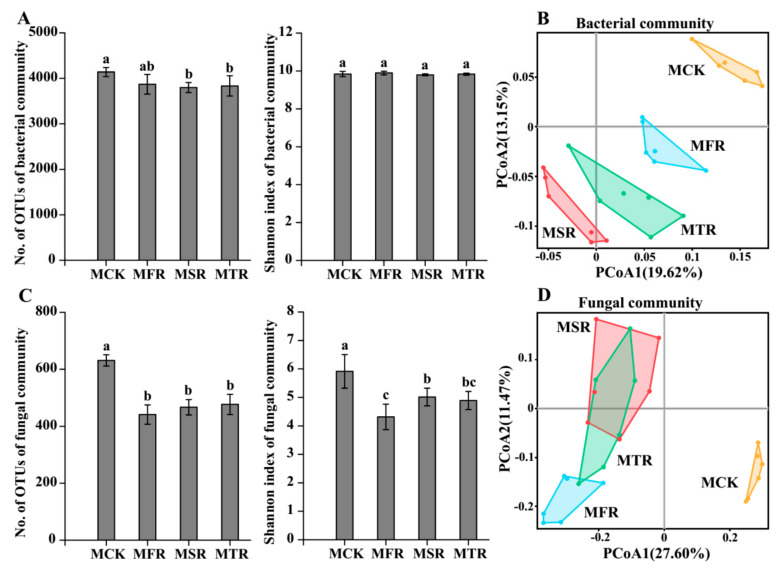
Alpha (**A**) and beta (**B**) diversities of soil bacterial communities and alpha (**C**) and beta (**D**) diversities of soil fungal communities. Beta diversities based on Bray–Curtis distance dissimilarity were visualized by principal coordinates analysis. The sequence similarity of OTUs was delineated at 97%. MCK represents no straw addition; MFR, MSR, and MTR represent one-year, two-year and three-year addition of rice straw. Different letters represent significant differences (*p* < 0.05, Tukey’s HSD test).

In the analysis of alpha diversity indices, the two-year and three-year addition treatments significantly decreased the observed OTU number of bacteria compared to the control (*p* < 0.05) (Figure 2A). Compared with the control, the rice straw treatment significantly decreased the observed OTU number and Shannon index of fungi (*p* < 0.05) (Figure 2C).

In the analysis of beta diversity, PCoA analysis of bacterial and fungal communities revealed that samples of the same treatment were grouped together, and there was a clear difference between the straw-treated samples and the control (bacterial community: ANOSIM, R = 0.74, *p* = 0.001; Adonis, R2 = 0.35, *p* = 0.001; fungal community: ANOSIM, R2 = 0.65, *p* = 0.001; Adonis, R2 = 0.47, *p* = 0.001) (Figure 2B,D).

### 3.6. Compositions of Bacterial and Fungal Communities in the Soils

**Figure 3:**
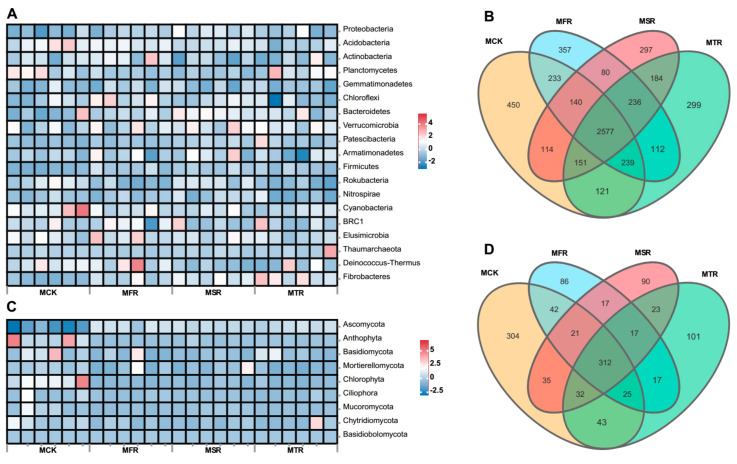
Heatmap of relative abundances at the phylum level for bacteria (**A**) and fungi (**B**), and Venn diagram analyses of OTUs for bacteria (**C**) and fungi (**D**). The rows were normalized with the Z-score normalization algorithm in the heatmap. MCK represents no straw addition; MFR, MSR, and MTR represent one-year, two-year and three-year addition of rice straw, respectively.

The Miseq sequencing data were classified at the 97% similarity level, including 32 bacterial phyla and 13 fungal phyla. There were 19 phyla with a relative abundance >1% at the bacterial phylum community level, and the relative abundance of bacterial phyla varied in the different treatments (Figure 3A and Supplementary Table S2). Compared with the control, the two-year treatment showed a higher relative abundance of Proteobacteria, and the relative abundance of Acidobacteria in the three-year treatment was lower (*p* < 0.05). The relative abundances of Bacteroidetes, Verrucomicrobia and Patescibacteria in the rice straw treatments were higher than in the control, albeit not significantly (*p* < 0.05). For the fungal phylum community, there were 9 phyla with a relative abundance >0.1% (Figure 3C and Supplementary Table S3). The dominant phyla were Ascomycota and Anthophyta, which total abundance >85%. The relative abundance of Ascomycota in rice straw treatments was significantly higher than in the control, and the relative abundances of Anthophyta and Chlorophyta in straw treatments were significantly lower than in the control (*p* < 0.05).

Among all samples, the control had the highest number of unique OTUs (450), and the two-year addition had the lowest number of unique OTUs (297) for bacteria (Figure 3B). For fungi, the control had the highest number of unique OTUs (304) and the one-year addition had the lowest number of unique OTUs (86) (Figure 3D).

**Figure 4:**
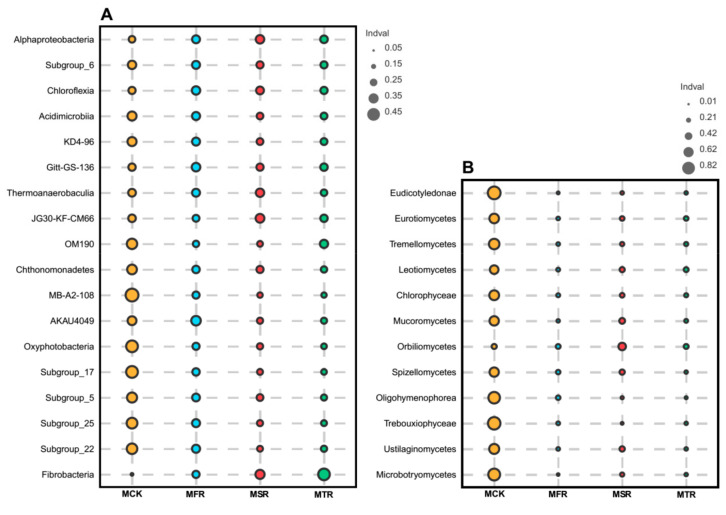
The indicator analysis at the class level for bacteria (**A**) and fungi (**B**). MCK represents no straw addition; MFR, MSR, and MTR represent one-year, two-year and three-year addition of rice straw, respectively.

At the class level, 53 bacterial and 25 fungal taxa were detected with a relative abundance >0.1% and the relative abundances of bacterial and fungal classes changed varied in the different treatments. For the bacterial class community, there were 18 bacterial taxa identified by indicator analysis; the relative abundances of most indicator bacterial classes in rice straw treatments were reduced compared with the control, but the relative abundances of Alphaproteobacteria, Chloroflexia, and Fibrobacteria were higher than in the control (Figure 4A and Supplementary Table S4). In the fungal class community, there were 12 fungal taxa identified by indicator analysis; the relative abundances of indicator fungal classes in rice straw treatments were lower than in the control, except for the relative abundance of Orbiliomycetes in MFR (Figure 4B and Supplementary Table S5).

**Figure 5:**
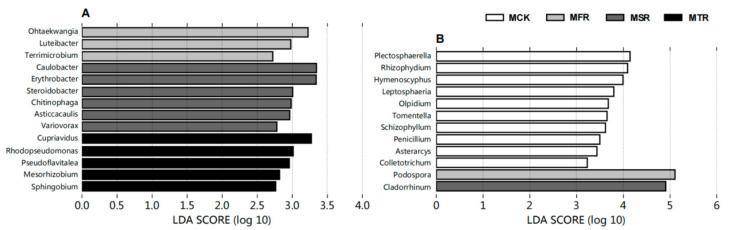
The LEfSe analysis at the genera for bacterial (**A**) and fungal (**B**). MCK represents no straw addition; MFR, MSR, and MTR represent one-year, two-year and three-year addition of rice straw, respectively.

LEfSe analysis was used to further evaluate the correlation between bacteria and fungi in the Chinese cabbage soil (average relative abundances > 0.1%) (Figure 5). The linear discriminant analysis (LDA) histogram scores showed that the control had no abundant genera in the bacterial community, but the control resulted in more abundant genera in the fungal community. In all treatments, 14 bacterial genera and 12 fungal genera were included in the differential microorganisms; the obtained genera were used for further investigation.

For the candidate bacterial genus communities, compared with the control, the straw treatments significantly increased the relative abundances of *Caulobacter*, *Steroidobacter*, *Ohtaekwangia*, *Mesorhizobium*, *Rhodopseudomonas*, *Erythrobacter*, and *Asticcacaulis*; the three-year treatment significantly increased the relative abundances of *Cupriavidus*, *Luteibacter*, *Sphingobium*, and *Pseudoflavitalea* (*p* < 0.05) (Supplementary Table S6).

For the candidate fungal genus communities, the straw treatment significantly reduced the relative abundances of *Penicillium* and *Colletotrichum* (*p* < 0.05). Compared with the control, the two-year and three-year treatments significantly increased the relative abundance of *Cladorrhinum*, and the one-year and three-year treatments significantly increased the relative abundance of *Podospora* (*p* < 0.05) (Supplementary Table S7).

### 3.7. Relationships between Soil Chemical Properties and Soil Microbial Communities

**Figure 6:**
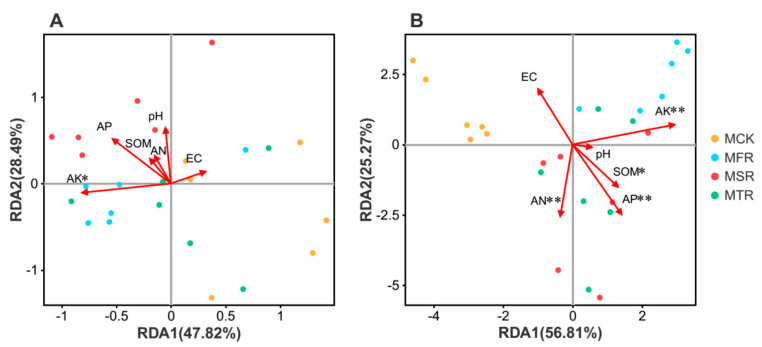
Redundancy analysis (RDA) of the relationship between the bacterial (**A**) and fungal (**B**) community structures and environmental variables in straw addition treatments. The environmental variables with statistical significance are represented by arrows. MCK represents no straw addition; MFR, MSR, and MTR represent one-year, two-year and three-year addition of rice straw. SOM, AN, AP, AK and EC represent soil organic matter, inorganic nitrogen, available phosphorus, available potassium and electrical conductivity, respectively. * *p* < 0.05; ** *p* < 0.01.

Redundancy analysis (RDA) was adopted to analyze the relationship between the environmental factors and the changes in the bacterial and fungal community structures among the different treatments. The results of the Mantel test showed that changes in the bacterial community structure were related to soil available phosphorus (r = 0.3091, *p* = 0.032) (Figure 6A), while soil organic matter (r = 0.3129, *p* = 0.018), inorganic nitrogen (r = 0.4685, *p* = 0.002), available phosphorus (r = 0.6256, *p* = 0.001), and available potassium (r = 0.7834, *p* = 0.001) were the dominant factors that can drive changes in the soil fungal community structure (Figure 6B).

### 3.8. Correlation Analysis between Soil Microorganisms and Resting Spores

A Pearson correlation analysis was conducted between the OTUs with a relative abundance >0.2% and the abundance of *P. brassicae* resting spores (*p* < 0.05).

There were 26 bacterial OTUs related to the resting spores. OTU 7 (WD2101_soil_group), OTU 8 (WD2101_soil_group), OTU 13 (Actinobacteria), OTU 15 (*Marmoricola*), OTU 48 (Chloroflexi), OTU 56 (*RB41*), OTU 65 (WD2101_soil_group), OTU 66 (Acidobacteria), OTU 69 (*Stenotrophomonas*), and OTU 76 (WD2101_soil_group) were extremely significantly positively correlated with the resting spores (*p* < 0.01). OTU 16 (WD2101_soil_group) was extremely positively correlated with the resting spores (*p* < 0.05). OTU 18 (Gemmatimonadetes), OTU 33 (*Phenylobacterium*), OTU 38 (Caulobacter), OTU 55 (*Aridibacter*), OTU 61 (*Acidibacter*), OTU 63 (Fimbriimonadaceae), and OTU 72 (BIrii41) had an extremely significant negative correlation with resting spores (*p* < 0.01). OTU 1 (*Sphingomonas*), OTU 5 (Sphingomonadaceae), OTU 29 (Blastocatellaceae), OTU 40 (Gemmatimonadaceae), OTU 45 (*Lysobacter*), OTU 49 (Micromonosporaceae), OTU 58 (Acidobacteria), and OTU 71 (*Altererythrobacter*) had a significant negative correlation with resting spores (*p* < 0.01) (Table 3).

For the fungi, 16 OTUs had a correlation with the resting spores. OTU 1 (*Cladorrhinum*), OTU 10 (Hypocreaceae), OTU 13 (*Vigna*), OTU 19 (Chaetomiaceae), OTU 20 (*Mortierella*), OTU 21 (*Podospora*), OTU 32 (*Tausonia*), OTU 36 (*Schizothecium*), OTU 38 (*Kotlabaea*), OTU 44 (Ascomycota) and OTU 52 (Ascomycota) were extremely significantly positively correlated with the resting spores (*p* < 0.01). OTU 37 (Microascaceae) and OTU 49 (*Bolbitius*) had an obviously positive correlation with the resting spores (*p* < 0.05). OTU 68 (Cordycipitaceae) had an extremely significant negative correlation with resting spores (*p* < 0.01). OTU 15 (Ascomycota) and OTU 85 had a significant negative correlation with resting spores (*p* < 0.05) (Table 4).

### 3.9. Microbial Ecological Guilds in Soil

**Figure 7:**
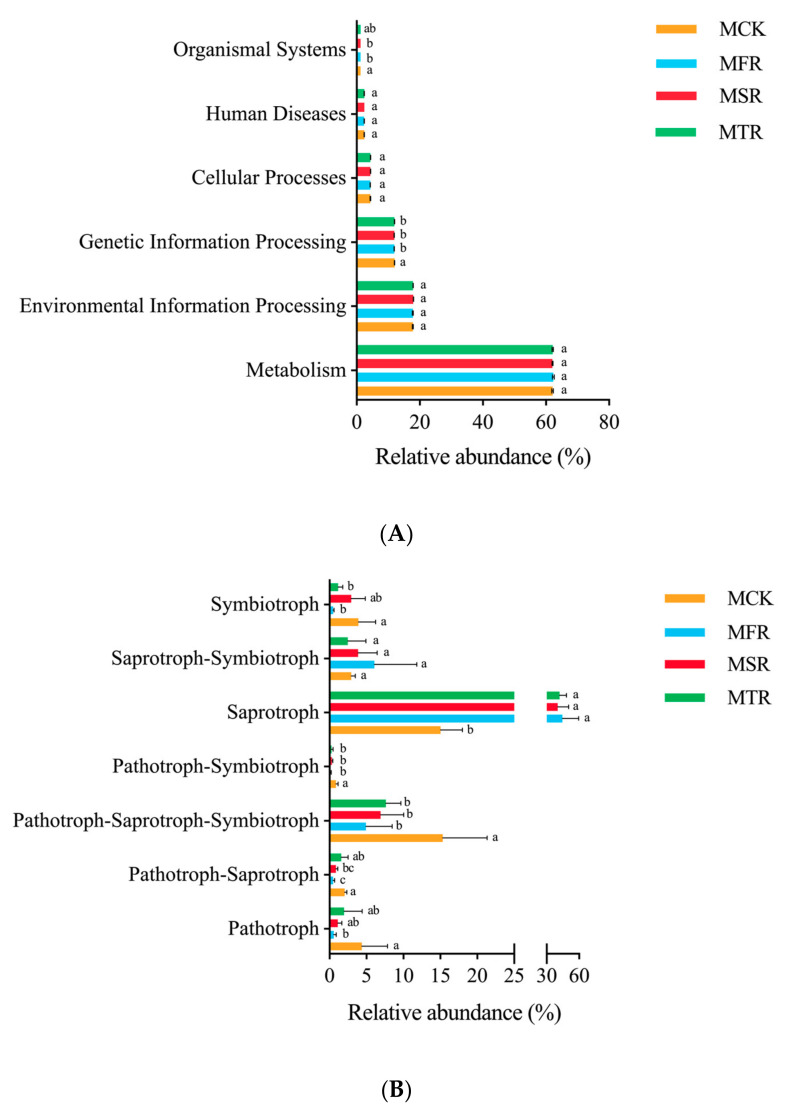
Soil bacterial (**A**) and fungal (**B**) functional prediction of soil in different treatments (Hierarchy Level 1). MCK represents no straw addition; MFR, MSR, and MTR represent one-year, two-year and three-year addition of rice straw. Different letters indicate significant differences (*p* < 0.05, Tukey’s HSD test).

There are 6 types of primary bacterial functional layers. Among these, MCK had the highest relative abundance of genetic information processing and organismal systems (*p* < 0.05) (Figure 7A).

For the fungi, seven primary functional layers were analyzed. Compared with the control, MFR and MTR had a lower relative abundance of symbiotrophs; MFR, MSR and MTR had a lower relative abundance of pathotroph-symbiotrophic and pathotroph-saprotroph-symbiotrophic fungi; the relative abundances of pathotroph-saprotrophic in MFR and MSR were significantly lower; and the relative abundance of pathotrophs in MFR was significantly lower than in MCK. In addition, the relative abundance of saprotrophs in MCK was significantly lower than in other treatments (*p* < 0.05) (Figure 7B).

## 4. Discussion

### 4.1. Effects of Straw Addition on the Growth of Chinese Cabbage

Previous studies have shown that straw return with appropriate management practices can promote plant growth and increase crop yield [19]. Our study found that, compared with no straw addition, rice straw addition promoted the growth of Chinese cabbage under prolonged experimental conditions for three years. As a typical environmentally friendly agricultural practice, crop straw addition can contribute to an increase in plant growth and crop yield [20]. Although many studies have found the negative effects of straw returning on crop production and yield, positive results can be achieved through the application of appropriate management practices. In cold black soil paddy fields, straw returning and controlled irrigation can achieve the goal of increasing the yield of rice fields [21].

### 4.2. Effects of Straw Addition on Soil Chemical Properties

Many studies have analyzed the influence of straw incorporation on crop yields and considered that sustainable crop production is supported by the balance of mineralization-related carbon loss in agricultural soil and improves a range of biological and physicochemical soil properties [22]. In addition, soil acidification is induced by reactive nitrogen inputs, and different nitrogen transformation processes contribute to different levels of acidification [23]. Our study found that the straw addition treatments reduced the content of available phosphorus and available potassium in soil, but the content of soil organic matter was less variable. This may be related to the specific regional ecological conditions and cropping systems, and the straw return method may have positive or negative effects on soil fertility and the environment [24]. For example, organic fertilizer instead of chemical fertilizer can alleviate soil acidification in vegetable fields, but the replacement ratio of organic fertilizer varies with different climatic conditions, initial soil properties, and management practices [25]. Meanwhile, this study found that the number of years of straw addition affects the extent of transformation of soil chemical properties. This result may be related to long-term organic input, as lignin-like plant residues increased the capacity of the soil organic carbon pool and potentially maintained the decomposability of soil organic carbon [26].

### 4.3. Effects of Straw Addition on Soil Microbial Communities

The return of straw not only affects the physical and chemical properties of soil but also affects the microbial community structure of bacteria and fungi. For example, straw returning to the field plays an important role in the cycle of the soil carbon pool; the accumulation of the soil organic carbon pool under conservation tillage is controlled by the complementary function of microbial and plant source components [27]. It was found that straw returning to the field could promote the growth of Chinese cabbage plants, inhibit the root disease of the Chinese cabbage, and increase the alpha diversity of Chinese cabbage rhizosphere bacterial community [10]. This experiment found that, compared with the control, the rice straw addition reduced some alpha diversity indices of bacteria and fungi, and the beta diversity indices of bacteria and fungi were affected obviously. This may be related to the “site effect”, as the microbial communities of richness and composition were distinguished by soil physicochemical characteristics, plant cover, and climate [28].

The local microbial activity of soil was stimulated after the addition of straw organic material, while microorganisms acquire nutrients by degrading complex organic compounds, and the composition and activity of soil microbial communities are important aspects of soil quality [29]. Meanwhile, the straw treatment increased the relative abundance of *Mesorhizobium* and *Rhodopseudomonas* but reduced the relative abundance of *Penicillium* and *Colletotrichum* in fungi. As beneficial microorganisms, *Mesorhizobium* strains have been shown to promote plant growth, not only increasing nutrient cycling but also suppressing pathogens [30]. As a plant growth-promoting rhizobacteria (PGPR), *Rhodopseudomonas* palustris in *Rhodopseudomonas* has been studied frequently; it can utilize a variety of substances as carbon and energy sources owing to its surprising metabolic versatility, suggesting that R. palustris could be used as a beneficial inoculant in agriculture [31]. Among the affected fungi, studies have found that *Penicillium* and *Colletotrichum* participate in the postharvest deterioration and rotting of tubers [32]. PGPR is a biocompatible approach involving inoculation, which can be used to decrease negative environmental impacts, especially in soils with continued use of chemical fertilizers. It is necessary to further study the beneficial and harmful strains of Chinese cabbage growth and the occurrence of Chinese cabbage disease in the future.

### 4.4. Effects of Straw Addition on the Clubroot of Chinese Cabbage

Soil-borne diseases are an important part of vegetable diseases, and the potential of straw addition to enhance soil nutrient contents and suppress soil-borne diseases has also been reported in recent years. For example, the addition of maize straw is an effective strategy to alleviate Phytophthora disease in monoculture pepper [20]. This experiment found that rice straw addition decreased the clubroot incidence rate, and the proportion of clubroot disease decreased with the increase in years of addition. Plant residues are the ultimate source of soil organic matter, but there is a continuum from relatively fresh plant tissue to highly evolved components [26]. The uncertain soil environmental changes impact the occurrence of soil-borne diseases persistently. According to the principle of plant–microbial interaction, there are two ways to control soil-borne diseases: to enhance the host’s resistance to pathogens or to reduce the invasion of pathogens [33]. Our study found that the straw addition reduced the abundance of *Plasmodiophora brassicae*, which can reduce the possibility of the host plant being infected. For studies of host resistance, previous studies have considered the indices of aboveground and belowground parts with different straw treatments, including the content of soluble sugar, soluble protein, ascorbic acid, nitrate, and root activity. However, no obvious rule was found in the seedling stage and harvest stage of Chinese cabbage, and a deeper study needs to be confirmed in further experiments.

There are many bacteria and fungi that are inversely correlated with resting spore abundance. OTU 1 belongs to *Sphingomonas*, which has the potential to inhibit the occurrence of plant diseases, such as the bacterial leaf blight of Christ’s thorn plants [34] and the powdery mildew and Fusarium head blight (FHB) of winter wheat [35]. OTU 33 belongs to *Phenylobacterium*, which has the effect of reducing bacterial wilt disease under continuous sesame cropping [36] and might have contributed to inhibiting the occurrence of replant disease of apple [37]. OTU 38, which belongs to *Caulobacter*, might have the potential effect of increasing the biomass of Arabidopsis, Citrullus, Zea mays and other species [38]. OTU 45, which belongs to *Lysobacter*, could produce a range of extracellular enzymes and other metabolites with activity against bacteria, fungi, oomycetes, and nematodes [39]. *Lysobacter* could produce antifungal compounds to inhibit *Plasmodiophora brassicae* resting spores and disease [40]. OTU 55 and OTU 61 both belong to *Aridibacter*. The enrichment of *Aridibacter* promotes denitrification processes [41], and *Aridibacter* was found to be associated with smut resistance in sugarcane [42]. Altererythrobacter participated in the process of inhibiting tomato Fusarium wilt disease [43].

For the fungi, the relative abundance of OTU 68 (Cordycipitaceae) had an extremely significant negative correlation with resting spores (*p* < 0.01). The Cordycepin, which belongs to Cordycipitaceae, has antifungal bioactivity [44].

## 5. Conclusions

In conclusion, the addition of rice straw promotes the growth of Chinese cabbage and controls the occurrence of root clubroot disease, and the soil chemical properties and microbial community structure were focused on. Regarding the chemical properties, the rice straw addition increased the content of available phosphorus and available potassium in soils. In addition, the results demonstrated that the addition of straw had an effect on both bacterial and fungal communities and was helpful for the management of clubroot, and the changes in bacteria and fungi were associated with soil available phosphorus. The soil microorganisms related to clubroot progression may give us insight into the study of the relationship between *P. brassicae* and soil microorganisms and the discovery of new perspectives for the bionomic control of clubroot disease.

## Figures and Tables

**Table 1:** Fresh weight and clubroot occurrence of Chinese cabbage.

	Fresh Shoot (kg/plant)	Incidence Rate of Clubroot (%)
MCK	0.43 ± 0.06 b	100.00 ± 0.00 a
MFR	1.07 ± 0.18 a	61.11 ± 4.81 b
MSR	1.23 ± 0.12 a	58.33 ± 8.33 b
MTR	1.09 ± 0.04 a	2.78 ± 0.481 c

MCK represents no straw addition; MFR, MSR, and MTR represent one-year, two-year and three-year addition of rice straw, respectively. Different letters indicate significant differences (*p* < 0.05, Tukey’s HSD test).

**Table 2:** Soil chemical properties’ response to straw application.

	EC (mS/cm)	pH	SOM (%)	AN (mg/kg)	AP (mg/kg)	AK (mg/kg)	TN (g/kg)
MCK	0.11 ± 0.00 a	7.39 ± 0.03 a	3.31 ± 0.26 a	133.88 ± 5.33 bc	720.93 ± 5.71 b	478.67 ± 10.07 c	2.06 ± 0.05 b
MFR	0.11 ± 0.00 a	7.44 ± 0.10 a	3.45 ± 0.34 a	121.37 ± 5.04 c	759.65 ± 24.34 b	592.00 ± 8.00 a	1.97 ± 0.05 b
MSR	0.11 ± 0.01 ab	7.43 ± 0.05 a	3.65 ± 0.09 a	149.94 ± 2.59 ab	856.85 ± 32.63 a	525.33 ± 9.24 b	2.22 ± 0.05 a
MTR	0.10 ± 0.00 b	7.38 ± 0.03 a	3.83 ± 0.23 a	155.86 ± 10.84 a	850.26 ± 12.44 a	544.00 ± 10.58 b	2.21 ± 0.06 a

EC, SOM, AN, AP, AK, and TN represent electrical conductivity, soil organic matter, alkaline hydrolytic nitrogen, available phosphorus, available potassium and total nitrogen, respectively. MCK represents no straw addition; MFR, MSR, and MTR represent one-year, two-year and three-year addition of rice straw, respectively. Values (mean ± SD) with different letters indicate significant differences at the 0.05 level (Tukey’s HSD test).

**Table 3:** Correlation analysis between resting spores and soil bacterial OTUs.

Bacterial OTUs (Relative Abundance in Soil %)	Correlation Coefficient with Resting Spores	Phylum	Family	Genus
OTU 7 (0.85)	0.583 **	Planctomycetes	WD2101_soil_group	Unclassified
OTU 8 (0.84)	0.607 **	Planctomycetes	WD2101_soil_group	Unclassified
OTU 13 (0.62)	0.588 **	Actinobacteria	Unclassified	Unclassified
OTU 15 (0.51)	0.682 **	Actinobacteria	Nocardioidaceae	*Marmoricola*
OTU 16 (0.59)	0.499 *	Planctomycetes	WD2101_soil_group	Unclassified
OTU 48 (0.30)	0.598 **	Chloroflexi	Unclassified	Unclassified
OTU 56 (0.26)	0.596 **	Acidobacteria	Pyrinomonadaceae	*RB41*
OTU 65 (0.22)	0.663 **	Planctomycetes	WD2101_soil_group	Unclassified
OTU 66 (0.25)	0.586 **	Acidobacteria	Unclassified	Unclassified
OTU 69 (0.35)	0.580 **	Proteobacteria	Xanthomonadaceae	*Stenotrophomonas*
OTU 76 (0.22)	0.534 **	Planctomycetes	WD2101_soil_group	Unclassified
OTU 1 (2.95)	−0.468 *	Proteobacteria	Sphingomonadaceae	*Sphingomonas*
OTU 5 (1.25)	−0.432 *	Proteobacteria	Sphingomonadaceae	Unclassified
OTU 18 (0.45)	−0.594 **	Gemmatimonadetes	Gemmatimonadaceae	Unclassified
OTU 29 (0.47)	−0.481 *	Acidobacteria	Blastocatellaceae	Unclassified
OTU 33 (0.40)	−0.628 **	Proteobacteria	Caulobacteraceae	*Phenylobacterium*
OTU 38 (0.33)	−0.785 **	Proteobacteria	Caulobacteraceae	*Caulobacter*
OTU 40 (0.26)	−0.450 *	Gemmatimonadetes	Gemmatimonadaceae	Unclassified
OTU 45 (0.23)	−0.436 *	Proteobacteria	Xanthomonadaceae	*Lysobacter*
OTU 49 (0.24)	−0.511 *	Actinobacteria	Micromonosporaceae	Unclassified
OTU 55 (0.26)	−0.548 **	Acidobacteria	Blastocatellaceae	*Aridibacter*
OTU 58 (0.23)	−0.443 *	Acidobacteria	Unclassified	Unclassified
OTU 61 (0.22)	−0.603 **	Proteobacteria	Unknown_Family	*Acidibacter*
OTU 63 (0.20)	−0.629 **	Armatimonadetes	Fimbriimonadaceae	Unclassified
OTU 71 (0.21)	−0.492 *	Proteobacteria	Sphingomonadaceae	*Altererythrobacter*
OTU 72 (0.23)	−0.689 **	Proteobacteria	BIrii41	Unclassified

“*” and “**” represent significant differences at the 0.05 and 0.01 probability levels, respectively, according to Pearson correlation analysis. “−” represents a negative correlation.

**Table 4:** Correlation analysis between resting spores and soil fungal OTUs.

Fungal OTUs (Relative Abundance in Soil %)	Correlation Coefficient with Resting Spores	Phylum	Family	Genus
OTU 1 (11.70)	0.835 **	Ascomycota	Lasiosphaeriaceae	*Cladorrhinum*
OTU 10 (2.06)	0.539 **	Ascomycota	Hypocreaceae	Unclassified
OTU 13 (3.05)	0.788 **	Anthophyta	Fabaceae	*Vigna*
OTU 19 (0.58)	0.595 **	Ascomycota	Chaetomiaceae	Unclassified
OTU 20 (1.02)	0.860 **	Mortierellomycota	Mortierellaceae	*Mortierella*
OTU 21 (0.36)	0.614 **	Ascomycota	Lasiosphaeriaceae	*Podospora*
OTU 32 (0.58)	0.545 **	Basidiomycota	Mrakiaceae	*Tausonia*
OTU 36 (0.41)	0.610 **	Ascomycota	Lasiosphaeriaceae	*Schizothecium*
OTU 37 (0.40)	0.483 *	Ascomycota	Microascaceae	Unclassified
OTU 38 (0.24)	0.727 **	Ascomycota	Pyronemataceae	*Kotlabaea*
OTU 44 (0.62)	0.665 **	Ascomycota	Unclassified	Unclassified
OTU 49 (0.51)	0.502 *	Basidiomycota	Bolbitiaceae	*Bolbitius*
OTU 52 (0.41)	0.519 **	Ascomycota	Unclassified	Unclassified
OTU 15 (1.27)	−0.477 *	Ascomycota	Unclassified	Unclassified
OTU 68 (0.38)	−0.601 **	Ascomycota	Cordycipitaceae	Unclassified
OTU 85 (0.27)	−0.444 *	Unclassified	Unclassified	Unclassified

“*” and “**” represent significant differences at the 0.05 and 0.01 probability levels, respectively, according to Pearson correlation analysis. “−” represents a negative correlation.

## Data Availability

Data will be made available on request.

## Supplementary Materials

The following supporting information can be downloaded at: https://www.mdpi.com/article/10.3390/microorganisms12040717/s1, Figure S1: The rarefaction curves of bacterial (A) and fungal (B) communities. Operational taxonomic units (OTUs) were delineated at 97% sequence similarity; Figure S2: The rarefaction curves of bacterial (A) and fungal (B) communities; Table S1: Basic soil chemical properties of the cultivated soil in July 2019; Table S2: Relative abundances of dominant bacterial phyla in soil; Table S3: Relative abundances of dominant fungal phyla in soil; Table S4: Relative abundances of dominant bacterial classes in soil; Table S5: Relative abundances of dominant fungal classes in soil; Table S6: Relative abundances of representative bacterial genera in soil; Table S7: Relative abundances of representative fungal genera in soil.
