# Supplementary material for: Application of Rice Straw Inhibits Clubroot Disease by Regulating the Microbial Community in Soil
Source: Microorganisms. 2024 Apr 1;12(4):717. doi: 10.3390/microorganisms12040717 (PMC11051980; doi:10.3390/microorganisms12040717)
Supplement: Supplementary file 1 [file microorganisms-12-00717-s001.zip › microorganisms-2851165-supplementary.pdf]

Article

Application of Rice Straw Inhibits Clubroot Disease by Regulating the Microbial Community in Soil

Zhe Han [1,2,†], Yiping Zhang [3,†], Chengqian Di [3], Hongwen Bi [1] and Kai Pan [3,*]

[1] Institute of Agricultural Remote Sensing and Information, Heilongjiang Academy of Agricultural Sciences, Harbin 150086, China; hanzhe6615@aliyun.com (Z.H.); bhw01@126.com (H.B.)
[2] Heilongjiang Academy of Agricultural Sciences Postdoctoral Program, Harbin 150086, China
[3] College of Horticulture and Landscape Architecture, Northeast Agricultural University, Harbin 150030, China; 18846054105@aliyun.com (Y.Z.); princedcq@sina.com (C.D.)
* Correspondence: mugonglin@aliyun.com; Tel.: +86-451-55190563
† These authors contributed equally to this work.

Supplementary Materials

Supplementary Figures

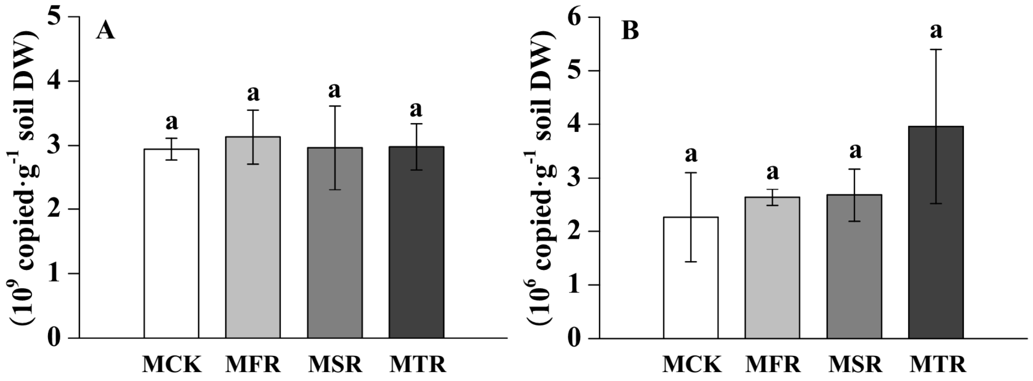

Figure S1. Abundance of bacterial (**A**) and fungal (**B**) in response to straw application. MCK represents no straw addition; MFR, MSR, and MTR represent one-year, two-year and three-year addition of rice straw. Error bars indicate the standard error, and different letters indicate significant differences at the 0.05 level (Tukey's HSD test).

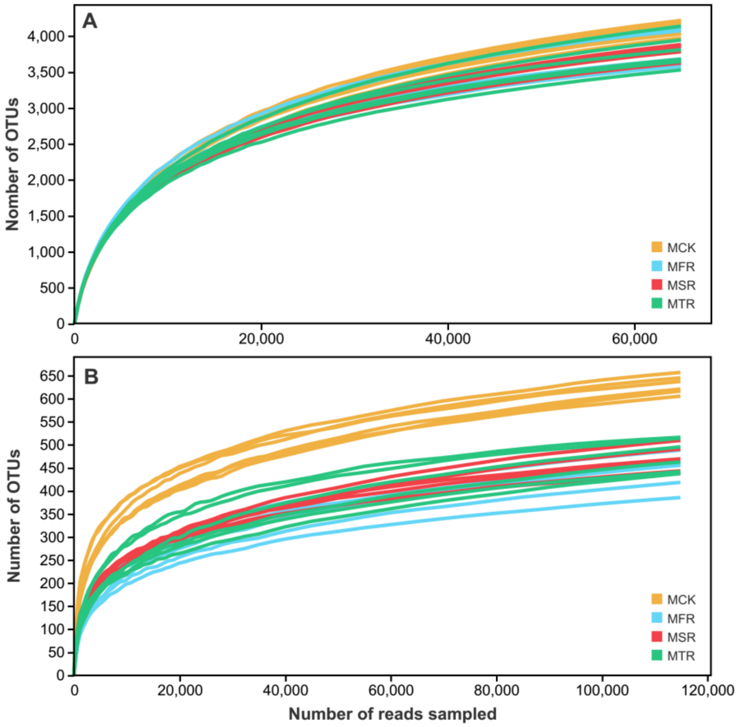

Figure S2. The rarefaction curves of bacterial (**A**) and fungal (**B**) communities. Operational taxonomic units (OTUs) were delineated at 97% sequence similarity. MCK represents no straw addition; MFR, MSR, and MTR represent one-year, two-year and three-year addition of rice straw.

Supplementary Tables

Table S1. Basic soil chemical properties of the cultivated soil in July 2019.

	**pH**	**EC (mS\cm)**	**SOM (%)**	**AN (mg\kg)**	**AP (mg\kg)**	**AK (mg\kg)**
MCK-MFR	7.15 ± 0.03a	0.127 ± 0.008b	3.10 ± 0.08b	144.36 ± 13.59b	764.67 ± 72.77b	513.33 ± 4.62c
MSR	6.84 ± 0.04b	0.143 ± 0.004ab	3.59 ± 0.28a	179.42 ± 15.25a	988.70 ± 5.50a	597.33 ± 2.31b
MTR	7.05 ± 0.11a	0.156 ± 0.008a	3.34 ± 0.16ab	145.51 ± 4.68b	1001.90 ± 2.08a	621.33 ± 8.33a

MCK-MFR, without straw addition in 2017 or 2018; MSR, straw addition once in 2018; MTR, straw addition both in 2017 and 2018. EC, electrical conductivity; SOM, soil organic matter; AN, available nitrogen; AP, available phosphorus; AK, available potassium. Values (mean ± SD) with different letters indicate significant differences at the 0.05 level (Tukey's HSD test).

Table S2. Relative abundances of dominant bacterial phyla in soil.

**Bacterial Phyla**	**MCK**	**MFR**	**MSR**	**MTR**
Proteobacteria	26.27 ± 2.32b	28.50 ± 1.58ab	30.94 ± 1.71a	28.47 ± 2.96ab
Acidobacteria	17.78 ± 2.09a	16.83 ± 0.59ab	15.80 ± 0.71ab	14.65 ± 1.57b
Actinobacteria	12.25 ± 0.94a	13.24 ± 1.38a	11.43 ± 1.11a	11.72 ± 1.49a
Planctomycetes	13.58 ± 4.73a	9.02 ± 1.33a	8.17 ± 1.30a	13.83 ± 4.89a
Gemmatimonadetes	9.02 ± 1.40a	9.60 ± 1.45a	9.19 ± 1.46a	8.81 ± 1.12a
Chloroflexi	8.06 ± 0.52ab	8.79 ± 0.65a	8.30 ± 0.4ab	7.73 ± 0.86b
Bacteroidetes	4.03 ± 1.72a	4.51 ± 0.46a	5.65 ± 0.56a	4.84 ± 1.03a
Verrucomicrobia	3.30 ± 1.02a	3.77 ± 1.05a	4.14 ± 1.01a	3.96 ± 0.71a
Patescibacteria	0.91 ± 0.14a	1.12 ± 0.13a	1.49 ± 0.21a	1.60 ± 0.91a
Armatimonadetes	0.93 ± 0.10a	1.01 ± 0.28a	1.10 ± 0.29a	0.90 ± 0.29a

MCK represents no straw addition; MFR, MSR, and MTR represent one-year, two-year and three-year addition of rice straw. Values (mean ± SD) with different letters indicate significant differences at the 0.05 level (Tukey's HSD test).

Table S3. Relative abundances of dominant fungal phyla in soil.

Fungal Phyla	MCK	MFR	MSR	MTR
Ascomycota	66.18 ± 8.09b	92.61 ± 4.77a	91.27 ± 1.74a	91.75 ± 2.63a
Anthophyta	14.67 ± 13.56a	0.50 ± 0.18b	1.42 ± 1.51b	1.34 ± 0.44b
Basidiomycota	4.70 ± 3.60a	2.57 ± 3.07a	1.75 ± 0.62a	3.03 ± 2.12a
Mortierellomycota	2.49 ± 0.41a	2.09 ± 1.93a	2.40 ± 1.96a	1.18 ± 0.48a
Chlorophyta	5.16 ± 2.70a	0.72 ± 0.13b	0.98 ± 0.37b	0.86 ± 0.41b
Ciliophora	1.42 ± 1.93a	0.46 ± 0.31a	0.45 ± 0.17a	0.33 ± 0.48a
Mucoromycota	0.31 ± 0.31a	0.04 ± 0.04b	0.14 ± 0.06ab	0.06 ± 0.02ab
Chytridiomycota	0.20 ± 0.11a	0.07 ± 0.06a	0.07 ± 0.04a	0.14 ± 0.23a

MCK represents no straw addition; MFR, MSR, and MTR represent one-year, two-year and three-year addition of rice straw, respectively. Values (mean ± SD) with different letters indicate significant differences at the 0.05 level (Tukey's HSD test).

Table S4. Relative abundances of dominant bacterial class in soil.

Bacterial Class	MCK	MFR	MSR	MTR
Alphaproteobacteria	11.11 ± 0.75c	13.84 ± 1.15ab	14.98 ± 0.95a	13.15 ± 0.68b
Subgroup_6	6.77 ± 0.94a	6.37 ± 0.37ab	5.55 ± 0.27b	5.54 ± 0.71b
Chloroflexia	3.53 ± 0.29b	4.26 ± 0.51a	3.91 ± 0.23ab	3.58 ± 0.42b
Acidimicrobiia	2.28 ± 0.23a	2.00 ± 0.24ab	1.58 ± 0.29c	1.63 ± 0.21bc
KD4-96	1.51 ± 0.10a	1.30 ± 0.09b	1.06 ± 0.03c	1.09 ± 0.11c
Gitt-GS-136	0.74 ± 0.10a	0.88 ± 0.10a	0.72 ± 0.10a	0.75 ± 0.13a
Thermoanaerobaculia	0.62 ± 0.07a	0.64 ± 0.06a	0.68 ± 0.06a	0.51 ± 0.07b
JG30-KF-CM66	0.31 ± 0.04ab	0.27 ± 0.06b	0.37 ± 0.06a	0.30 ± 0.06ab
OM190	0.43 ± 0.18a	0.24 ± 0.05b	0.21 ± 0.05b	0.33 ± 0.13ab
Chthonomonadetes	0.32 ± 0.08a	0.24 ± 0.09ab	0.22 ± 0.07ab	0.19 ± 0.08b
MB-A2-108	0.43 ± 0.05a	0.22 ± 0.05b	0.15 ± 0.05b	0.15 ± 0.05b
AKAU4049	0.26 ± 0.04a	0.28 ± 0.05a	0.16 ± 0.03b	0.16 ± 0.04b
Oxyphotobacteria	0.30 ± 0.15a	0.17 ± 0.07ab	0.14 ± 0.06b	0.13 ± 0.04b
Subgroup_17	0.21 ± 0.06a	0.12 ± 0.02b	0.09 ± 0.02b	0.09 ± 0.02b
Subgroup_5	0.15 ± 0.01a	0.11 ± 0.02b	0.09 ± 0.01bc	0.08 ± 0.02c
Subgroup_25	0.10 ± 0.02a	0.07 ± 0.01ab	0.05 ± 0.03b	0.05 ± 0.02b
Subgroup_22	0.10 ± 0.02a	0.07 ± 0.02b	0.05 ± 0.01b	0.05 ± 0.01b
Fibrobacteria	0.01 ± 0.01b	0.05 ± 0.02ab	0.06 ± 0.02a	0.08 ± 0.04a

MCK represents no straw addition; MFR, MSR, and MTR represent one-year, two-year and three-year addition of rice straw, respectively. Values (mean ± SD) with different letters indicate significant differences at the 0.05 level (Tukey's HSD test).

Table S5. Relative abundances of dominant fungal class in soil.

Fungal Class	MCK	MFR	MSR	MTR
Eudicotyledonae	14.67 ± 13.57a	0.50 ± 0.18b	1.42 ± 1.51b	1.33 ± 0.43b
Eurotiomycetes	3.78 ± 2.10a	0.61 ± 0.26b	1.11 ± 0.38b	1.10 ± 0.43b
Tremellomycetes	2.58 ± 2.55a	0.37 ± 0.15b	0.54 ± 0.21ab	0.53 ± 0.24ab
Leotiomycetes	0.76 ± 0.20a	0.16 ± 0.07b	0.32 ± 0.16b	0.29 ± 0.21b
Chlorophyceae	1.77 ± 0.68a	0.35 ± 0.10b	0.51 ± 0.24b	0.38 ± 0.17b
Mucoromycetes	0.29 ± 0.32a	0.04 ± 0.04a	0.14 ± 0.06a	0.06 ± 0.02a
Orbiliomycetes	0.11 ± 0.09a	0.10 ± 0.06a	0.22 ± 0.12a	0.10 ± 0.07a
Spizellomycetes	0.16 ± 0.13a	0.05 ± 0.04ab	0.06 ± 0.04ab	0.02 ± 0.02b
Oligohymenophorea	0.09 ± 0.06a	0.02 ± 0.02b	0.01 ± 0.01b	0.01 ± 0.01b
Trebouxiophyceae	0.12 ± 0.07a	0.02 ± 0.01b	0.00 ± 0.00b	0.01 ± 0.02b
Ustilaginomycetes	0.02 ± 0.03a	0.00 ± 0.00a	0.01 ± 0.01a	0.00 ± 0.00a
Microbotryomycetes	0.07 ± 0.09a	0.00 ± 0.00a	0.02 ± 0.02a	0.01 ± 0.01a

MCK represents no straw addition; MFR, MSR, and MTR represent one-year, two-year and three-year addition of rice straw. Values (mean ± SD) with different letters indicate significant differences at the 0.05 level (Tukey's HSD test).

Table S6. Relative abundances of representative bacterial genera in soil.

Bacterial genus	MCK	MFR	MSR	MTR
Caulobacter	0.14 ± 0.03c	0.41 ± 0.09b	0.56 ± 0.07a	0.44 ± 0.06b
Steroidobacter	0.15 ± 0.01b	0.28 ± 0.07a	0.32 ± 0.05a	0.26 ± 0.05a
Ohtaekwangia	0.06 ± 0.02b	0.38 ± 0.12a	0.33 ± 0.06a	0.30 ± 0.10a
Cupriavidus	0.03 ± 0.01b	0.28 ± 0.16ab	0.14 ± 0.04ab	0.40 ± 0.29a
Mesorhizobium	0.06 ± 0.01b	0.11 ± 0.04a	0.12 ± 0.01a	0.12 ± 0.02a
Rhodopseudomonas	0.04 ± 0.01b	0.09 ± 0.02a	0.09 ± 0.02a	0.09 ± 0.02a
Luteibacter	0.01 ± 0.01b	0.18 ± 0.11a	0.10 ± 0.05ab	0.13 ± 0.08a
Erythrobacter	0.03 ± 0.01c	0.05 ± 0.01b	0.07 ± 0.01a	0.04 ± 0.01b
Sphingobium	0.01 ± 0.00b	0.07 ± 0.05a	0.06 ± 0.03ab	0.08 ± 0.03a
Pseudoflavitalea	0.00 ± 0.00b	0.08 ± 0.02ab	0.05 ± 0.02b	0.16 ± 0.10a
Variovorax	0.01 ± 0.01b	0.06 ± 0.02ab	0.09 ± 0.06a	0.07 ± 0.03ab
Asticcacaulis	0.00 ± 0.00b	0.03 ± 0.02a	0.03 ± 0.01a	0.03 ± 0.01a

MCK represents no straw addition; MFR, MSR, and MTR represent one-year, two-year and three-year addition of rice straw. Values (mean ± SD) with different letters indicate significant differences at the 0.05 level (Tukey's HSD test).

Table S7. Relative abundances of representative fungal genera in soil.

Fungal genus	MCK	MFR	MSR	MTR
Cladorrhinum	2.88 ± 0.95b	8.64 ± 2.37ab	18.73 ± 8.98a	16.56 ± 9.21a
Podospora	1.51 ± 0.86c	27.37 ± 9.8a	12.39 ± 8.12bc	14.23 ± 5.72b
Plectosphaerella	2.89 ± 1.23a	0.37 ± 0.06b	2.89 ± 1.94a	0.88 ± 0.49b
Penicillium	0.62 ± 0.25a	0.06 ± 0.05b	0.09 ± 0.05b	0.12 ± 0.04b
Colletotrichum	0.23 ± 0.11a	0.00 ± 0.00b	0.02 ± 0.02b	0.01 ± 0.00b

MCK represents no straw addition; MFR, MSR, and MTR represent one-year, two-year and three-year addition of rice straw. Values (mean ± SD) with different letters indicate significant differences at the 0.05 level (Tukey's HSD test).
